# Effects of Suberoylanilide Hydroxamic Acid (SAHA) on the Inflammatory Response in Lipopolysaccharide-Induced N9 Microglial Cells

**DOI:** 10.7759/cureus.32428

**Published:** 2022-12-12

**Authors:** Zülfinaz Betül Çelik, Caner Günaydın

**Affiliations:** 1 Medical Biology, Samsun University Faculty of Medicine, Samsun, TUR; 2 Pharmacology, Samsun University Faculty of Medicine, Samsun, TUR

**Keywords:** cytokine, neuroinflammation, n9 microglia, lipopolysaccharide, histone deacetylation

## Abstract

Introduction: Epigenetics has shown promising results for understanding the different behaviors of microglia under the context of neuroinflammation. However, to our knowledge, the results of this complex mechanism with novel pharmacological agents such as histone deacetylase inhibitors (HDACis) are still missing. In this study, we aimed to investigate the effects of suberoylanilide hydroxamic acid (SAHA), a pan-HDACi, on the lipopolysaccharide (LPS)-induced neuroinflammation model in the N9 microglial cells.

Methods: Microglial cells were treated with SAHA (0.25, 0.5, 1.0, 1.25, 1.5 µM) and LPS (100 ng/mL) for 24 hours. Then, levels of the pro/anti-inflammatory cytokines interleukin-1 beta (IL-1β), IL-6, tumor necrosis factor alpha (TNF-α), and IL-10 were determined by the enzyme-linked immunosorbent assay. The total cellular HDAC activity was determined by colorimetric analysis. Additionally, the expression levels of nuclear factor kappa-B (NF-κB) were quantified via western blotting.

Results: SAHA (1.0 and 1.25 µM) attenuated the LPS-induced inflammatory response of microglial cells via decreasing NF-κB expression and pro-inflammatory cytokines (IL-1β, IL-6, TNF-α) in the N9 microglial cells. Moreover, SAHA treatment improved IL-10 levels and prevented the LPS-induced increase in the HDAC activity in the microglial cells.

Conclusion: Our results suggest SAHA attenuates the LPS-induced inflammatory response in the N9 microglial cells, and regulation of histone acetylation with HDACis might be a rational approach for the treatment of neuroinflammation.

## Introduction

The role of neuroinflammation and its interplay with central nervous system (central nervous system) disorders, especially neurodegenerative diseases, has gained significant attention in recent years. After the mechanisms that might affect the start and progression of neuroinflammation, different factors entered the research spectrum for further investigations. Microglia are the immune system anchors of the CNS and are currently accepted as the primary regulator for inflammatory responses and neuroinflammatory pathogenesis [1]. Therefore, the comprehension of the role of the microglia under inflammatory circumstances is crucial for finding therapeutic options and exploring possible targets in neurodegeneration. It has been demonstrated that microglia cells activate upon acute CNS injury [2]. However, the regulation of microglia in chronic conditions such as neuroinflammation has been thought of as a neuroprotective behavior [3]. Mechanisms behind these responses are not completely described; cytokines such as interleukin-1 beta (IL-1β) and tumor necrosis factor alpha (TNF-α) and proteins such as nuclear factor kappa B (NF-κB) have been referred to in several reports [4]. However, these claimed long-term effects of microglia and its activation have been further linked with chronic cytokine production and harmful effects on the CNS [5]. Although the chronic activation of microglia and its epigenetic modification are mostly studied in brain tumors, reports about microglia and its relationship with epigenetics shift attention to neurodegenerative disorders.

Histone modification, particularly histone acetylation, is a widely studied topic and has been demonstrated as responsible for the neuroregulatory response [6]. Histone modifiers such as histone deacetylase inhibitors (HDACis) showed up as neuroprotective agents in several experimental models [7]. HDAC inhibition has already been shown to reduce β-amyloid toxicity and protect dopaminergic neurons by inhibiting the NF-κB pathway [8]. Therefore, investigating and clarifying the actions of HDAC inhibitors in microglial cells are vital in the context of coping with chronic neuroinflammation.

Current evidence suggests that SAHA, which is a pan-HDACi, has potent anti-inflammatory and immunomodulatory actions [9]. SAHA also enhances acetylation levels and regulates the non-histone proteins epigenetically, which include transcription factors related to inflammation such as signal transducer and activator of transcription-3 (STAT3) and NF-κB [10]. Growing evidence demonstrates that SAHA has remarkable immunoregulatory effects in vivo and in vitro. Later studies validate these immunoregulatory and anti-inflammatory effects in animal models of rheumatoid arthritis, inflammatory bowel disease, lupus nephritis, hepatitis, and graft versus host disease [11].

Lipopolysaccharide (LPS) is known as a potent stimulant that mimics neuroinflammation via toll-like receptor 4 in microglia, which can lead to neuronal death. Inhibiting the LPS-induced microglial activation relies on the repression of the NF-κB activation. Moreover, the dysregulation of acetylation has been associated with neuroinflammation and neurodegeneration-related death of neurons [12]. Although an LPS-induced increase in the histone deacetylation activity was demonstrated in several reports, effects on histone deacetylase inhibitors in the LPS-induced neuroinflammation still have shortcomings [13]. The research information mentioned about the effects of SAHA persuaded us to investigate the possible actions of histone acetylation in the N9-microglial cells and clarify the effects of SAHA on NF-κB signaling and related cytokines and histone deacetylase activity. Therefore, in this study, we aimed to investigate the effects of SAHA and histone acetylation on the LPS-induced neuroinflammation in the N9 microglial cells by determining the levels of the pro/anti-inflammatory cytokines (IL-1β, IL-6, TNF-α, and IL-10), NF-κB, and the total HDAC activity.

## Materials and methods

Chemicals and reagents

The following reagents were used in the study: SAHA (Sigma-Aldrich, USA), LPS (B5:O55; Sigma-Aldrich), dimethylsulfoxide (DMSO; Sigma-Aldrich), 3-(4,5-dimethylthiazol-2-yl)-2,5-diphenyl tetrazolium bromide (MTT; Sigma-Aldrich), fetal bovine serum (FBS; Gibco BRL, USA), Leibovitz’s L-15 medium (Gibco BRL), bovine serum albumin (BSA; Gibco BRL), trypsin-ethylenediaminetetraacetic acid, or trypsin-EDTA (0.25% and 0.0025%; Gibco BRL), Dulbecco’s Modified Eagle Medium (DMEM; Gibco BRL), L-glutamine (Gibco BRL), and Dulbecco’s Phosphate-Buffered Saline (dPBS; Gibco BRL). Rabbit polyclonal anti-NF-κB (ab16502; Abcam, UK), rabbit polyclonal β-actin (ab8227; Abcam), and goat anti-rabbit IgG (H+L)-horseradish peroxidase (HRP) conjugate (#1706515; Bio-Rad, USA) were used for primary and secondary antibody incubation. LPS was dissolved in sterile water, and SAHA was dissolved in DMSO with the caution of toxicity (less than 0.001%).

Cell culture

The N9 microglial cell line (passage number 10, accession number CVCL_0452; Shanghai Si-Xin Biotechnology Ltd., Shanghai, China) was kindly donated by Cellular Pharmacology Center, Italy. N9 microglial cells were cultured in the RPMI 1640 medium (supplemented with 10% FBS, 2 mM L-glutamine, and 1% penicillin/streptomycin) at 37°C in a humidified atmosphere containing 5% CO_2_.

Analysis of cell viability

Cell viability was assessed with the MTT test. The cells were seeded to plates at 10^4^ cells per well [14]. Cells were maintained in plates for overnight incubation. After the incubation period, cells were treated with SAHA (0.25, 0.5, 1.0, 1.25, and 1.5 µM), LPS (100 ng/ mL), and SAHA+LPS simultaneously for 24 hours; then, culture mediums were discarded and the MTT (5 mg/mL) solution was added for the incubation period. At the end of the four-hour incubation period in the dark at 37°C, the MTT solution was aspirated and cells were rinsed with DMSO (100 µL) to dissolve the formed formazan crystals. The optic density of cells was determined with the microplate reader at 550 nm wavelength (Tecan, Switzerland).

Determination of TNF-α, IL-6, IL-1β, and IL-10 levels

Following the drug treatment period, mediums were aspirated for the measurement of IL-6 (ab100712; Abcam), IL-1β (MLB00C; R&D Systems, USA), TNF-α (ADI-900-047; Enzo Life Sciences, USA), and IL-10 (E04594m; Cusabio Biotech, UK) levels with the enzyme-linked immunosorbent assay (ELISA) method. Expression levels were determined from 100 µL mediums of all treatments by strictly following the manufacturer’s protocols.

Protein extractions

Cell lysates were prepared from six-well plates. After drug treatments, all wells were washed with the radioimmunoprecipitation assay (RIPA) buffer (50 mM Tris, pH 8.0, 150 mM sodium chloride, 0.5% sodium deoxycholate, 0.1% sodium dodecyl sulfate, or SDS, 1.0% NP-40) supplemented with SIGMAFAST® protease inhibitor and cells were scraped. The total protein quantity of samples was measured with the bicinchoninic acid (BCA) protein assay kit according to the manufacturer’s instructions (#SK3021; Bio Basic, Canada). Samples employed in the western blot analysis were treated with the sample buffer (5% 2-mercaptoethanol, 4% SDS, 20% glycerol, 0.125 M Tris HCl, 0.004% bromophenol blue) and reduced at 95°C for five minutes.

Western blot analysis

After the determination of the protein contents of the samples, equal amounts of samples were loaded into 4%-20% SDS-polyacrylamide gel electrophoresis (SDS-PAGE) [15]. Following separation, the proteins were transferred to polyvinylidene fluoride (PVDF) membranes with a semi-dry blotting device (Bio-Rad, USA). The membranes were blocked with a 5% BSA solution to prevent non-specific binding. The membranes were washed with 0.01% Tris-buffered saline-Tween 20 (TBS-T) three times, and then incubated with primary antibodies (β-actin and NF-κB) overnight at +4°C. Next, the washing process was repeated, and membranes were incubated with HRP-conjugated secondary antibody for one hour; then the electrochemiluminescence (ECL) substrate was added for visualization. Membranes were visualized in ChemiDoc® (Bio-Rad), and band intensities were measured with ImageJ software (free software; from National Institutes of Health). The intensity was normalized to the β-actin ratio.

Total HDAC activity

After determining the protein concentration of each cell lysate, the total HDAC activity was determined by the histone deacetylase-activity assay kit (#GTX85529; Genetex, USA) colorimetrically; 50-100 µg protein per sample was sonicated, incubated, and treated with Boc-Lys(Ac)-pNA (HDAC substrate) for at least 30 minutes at 37°C. Then, the lysine developer, supplemented with the kit, was added and the reaction was stopped. The HDAC activity was measured by optic densities at 405 nm (Tecan, Switzerland).

Statistical analysis

Statistical analysis was performed with IBM SPSS Statistics for Windows, Version 28.0 (IBM Corp., Armonk, NY). Following the determination of data distribution, the one-way analysis of variance (ANOVA) test was performed. For multiple comparisons, Tukey’s post hoc test was used. P values less than 0.05 were assessed as significant.

## Results

Inhibition of the LPS-induced decrease in cell viability by SAHA in N9 microglial cells

**Figure 1:**
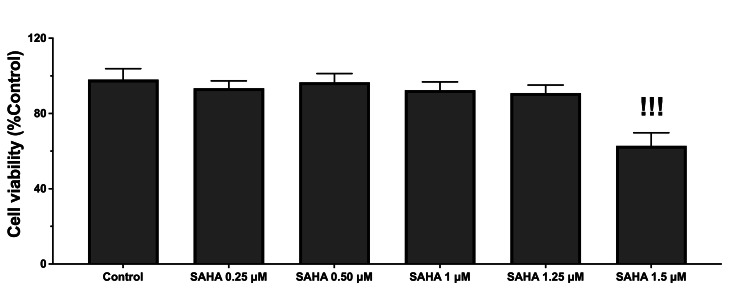
Cellular viability assays of single SAHA treatment at 0.25, 0.50, 1.0, 1.25, and 1.5 µM concentrations SAHA: suberoylanilide hydroxamic acid Only the 1.5 µM concentration of SAHA significantly decreased cellular viability. Data are expressed as means ± SDs of at least six replicates. ^!!!^P<0.001 versus control

**Figure 2:**
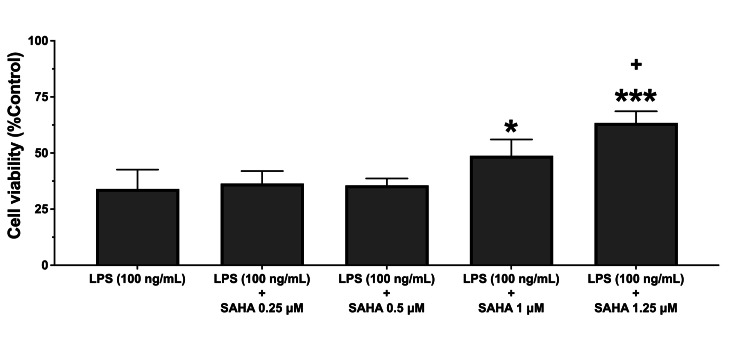
Cell viability results of the MTT test MTT: 3-(4,5-dimethylthiazol-2-yl)-2,5-diphenyl tetrazolium bromide; SAHA: suberoylanilide hydroxamic acid; LPS: lipopolysaccharide Effects of SAHA (0.25, 0.5, 1.0, 1.25, and 1.5 µM) on LPS (100 ng/mL)-induced decrease in cellular viability are shown. SAHA (1.0and 1.25 µM) significantly prevented the LPS-induced decrease in the cellular viability in the microglial cells. Data are expressed as means ± SDs of at least six replicates. ***P<0.001 and *P<0.05 versus LPS (100 ng/mL) ^+^P<0.05 versus LPS (100 ng/mL) + SAHA (1 µM)

MTT test results showed that single SAHA treatment at the concentrations of 0.25, 0.5, 1.0, and 1.25 µM did not affect cellular viability (P>0.05; Figure 1). However, SAHA at the concentration of 1.5 µM significantly reduced cell survival in the N9 microglial cells (P<0.001; Figure 1). Nevertheless, SAHA, at 1.0 µM and 1.25 µM concentrations, significantly increased cell viability in LPS-treated microglial cells (P<0.001 and P<0.001, respectively; Figure 2). Moreover, SAHA at a concentration of 1.25 µM significantly increased cell viability in the N9 cells compared to 1.0 µM concentration (P=0.021; Figure 2).

Prevention of LPS-induced TNF-α, IL-6, IL-1β increases, and IL-10 decrease by SAHA treatment

**Figure 3:**
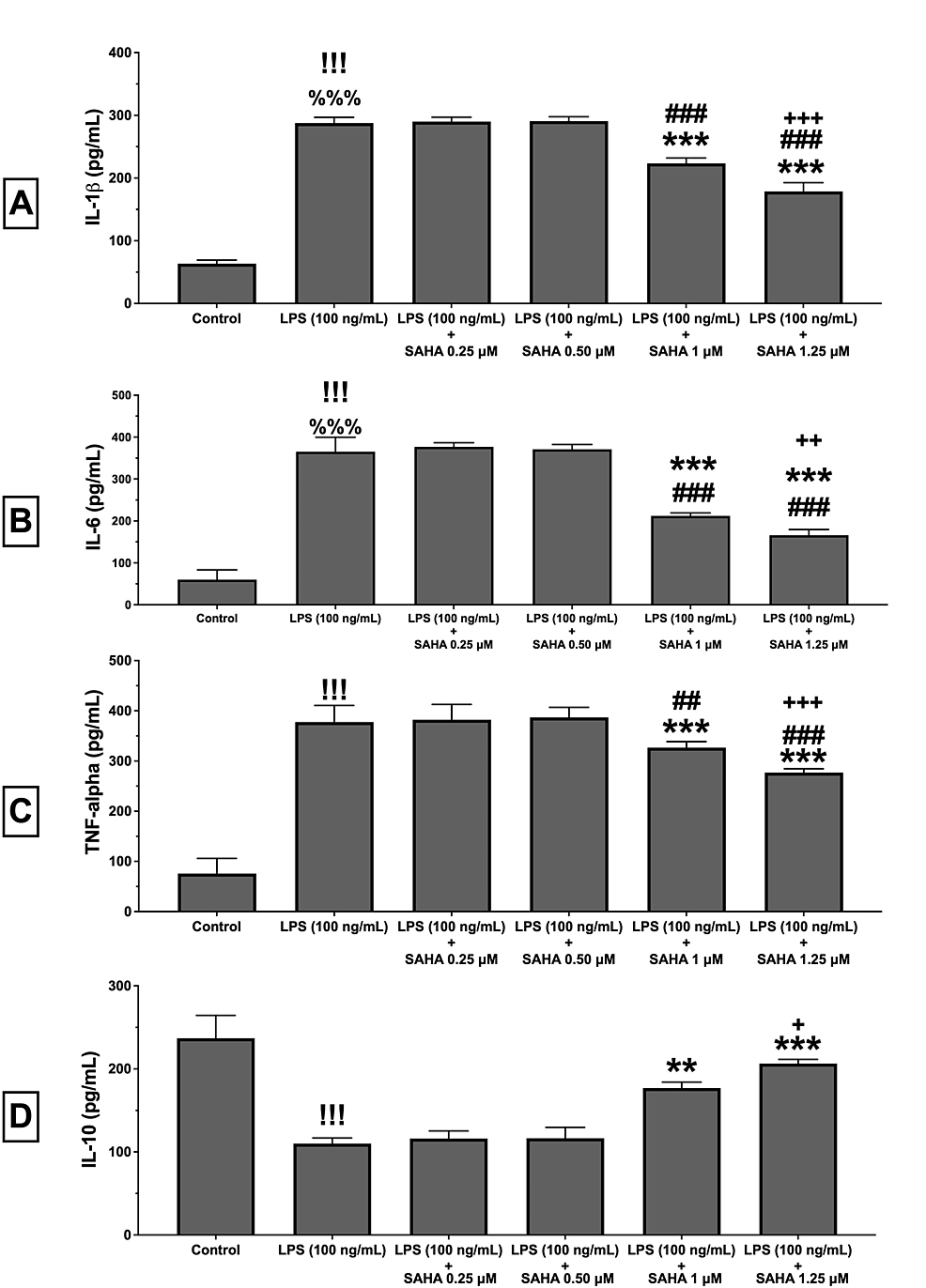
Cytokine levels determined by ELISA SAHA: suberoylanilide hydroxamic acid; LPS: lipopolysaccharide; IL-1β: interleukin-1 beta; IL-6: interleukin-6; IL-10: interleukin-10; TNF-α: tumor necrosis factor alpha; ELISA: enzyme-linked immunosorbent assay Effects of SAHA (0.25, 0.5, 1.0, 1.25 µM) on IL-1β (A), IL-6 (B), TNF-α (C), and IL-10 (D) levels in LPS-induced cells are demonstrated here. SAHA (1.0 and 1.25 µM) significantly attenuated LPS-induced increase in the IL-1β, IL-6, TNF-α levels. Additionally, SAHA (1.0 and 1.25 µM) prevented the decrease in the IL-10 levels. There was a significant difference between 1.0 and 1.25 µM concentrations of SAHA treatment in the N9 microglial cells. Data are expressed as means ± SDs of at least six replicates. ^!!!^P<0.001 and ^%%%^P<0.001 versus control ***P<0.001, **P<0.01 and ^###^P<0.001 ^##^P<0.01 versus LPS (100 ng/mL) ^+++^P<0.001, ^++^P<0.01, ^+^P<0.05 versus LPS (100 ng/mL) + SAHA (1 µM)

A biochemical analysis was performed for the measurement of cytokine levels. Our results demonstrated that single LPS treatment significantly increased IL-1β, IL-6, and TNF-α levels in the N9 microglial cells, as expected (P<0.001 for all; Figure 3A, Figure 3C). Additionally, it also significantly decreased IL-10 levels in the microglial cells (P<0.001; Figure 3D). However, SAHA at concentrations of 1.0 and 1.25 µM significantly ameliorated the LPS-induced increase in IL-1β, IL-6, and TNF-α levels (P<0.001 and P=0.002, respectively; Figure 3A, Figure 3C). Furthermore, SAHA at concentrations of 1.0 and 1.25 µM significantly prevented the LPS-induced decrease in the IL-10 levels in the microglial cells (P<0.001 and P=0.01; Figure 3D). Moreover, SAHA treatment in LPS-induced microglial cells showed significant differences between 1.0 and 1.25 µM concentrations in these parameters (P<0.001 for IL-1β, P=0.006 for IL-6, P=0.008 for TNF-α and P=0.032 for IL-10; Figure 3D).

Amelioration of the LPS-induced cytoplasmic NF-κB expression increase by SAHA

**Figure 4:**
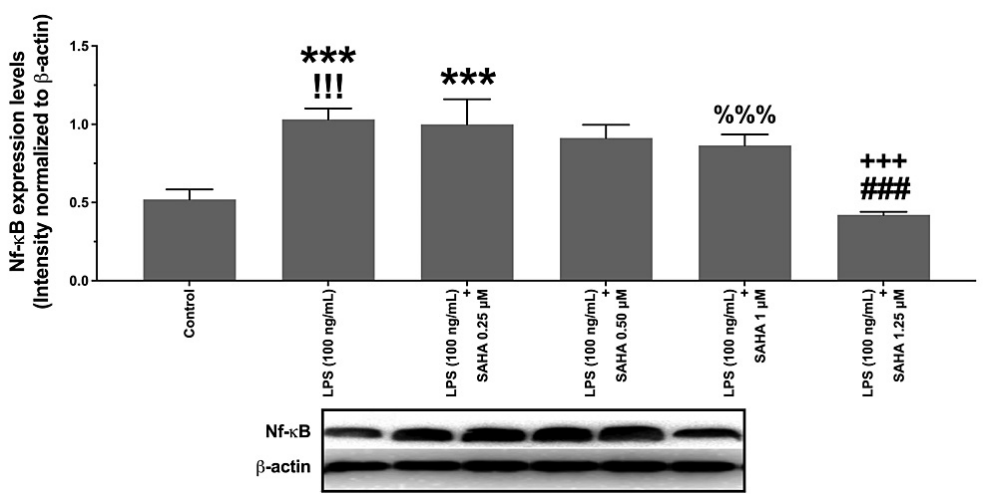
Western blot analysis results and the effects of SAHA (0.25, 0.5, 1.0, 1.25 µM) on NF-κB expression levels in LPS-induced microglial cells SAHA: suberoylanilide hydroxamic acid; LPS: lipopolysaccharide; NF-κB: nuclear factor kappa-B SAHA, at the 1.25 µM concentration, significantly ameliorated NF-κB expression levels in the N9 microglial cells. Data are expressed as means ± SDs, and blot images are representative of three independent samples (n=3). ^!!!^P<0.001 and ^%%%^P<0.001 versus control ***P<0.001 and ^###^P<0.001 versus LPS (100 ng/mL) ^+++^P<0.001 versus LPS (100 ng/mL) + SAHA (1 µM)

Western blot analysis results exhibited that NF-κB expression was increased in the N9 microglial cells after LPS treatment, as expected (P<0.001; Figure 4). Furthermore, SAHA (at 1.25 µM) mitigated LPS-induced NF-κB expression in the microglial cells (P<0.001; Figure 4), and this was coherent with the TNF-α, IL-6, IL-1β, and IL-10 level change (P>0.05; Figure 4). The western blot image is provided in the appendix.

Inhibition of the LPS-induced increase in the total HDAC activity by SAHA

**Figure 5:**
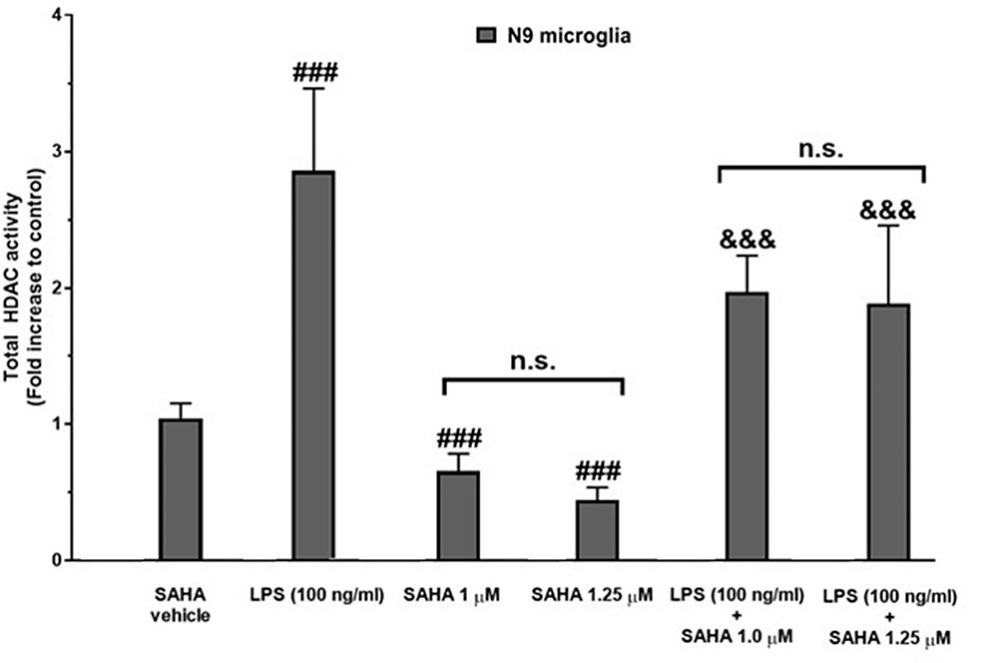
Results of the total HDAC activity assay SAHA: suberoylanilide hydroxamic acid; LPS: lipopolysaccharide; HDAC: histone deacetylase; n.s: not significant LPS increased the total HDAC activity. SAHA treatment at concentrations of 1.0 and 1.25 µM decreased the total HDAC activity. Additionally, SAHA prevented the increase in the LPS-induced total HDAC activity in all doses. Data are expressed as means ± SDs of at least six replicates. ^###^P<0.001 versus SAHA vehicle ^&&&^P<0.001 versus LPS (100 ng/mL)

**Figure 6:**
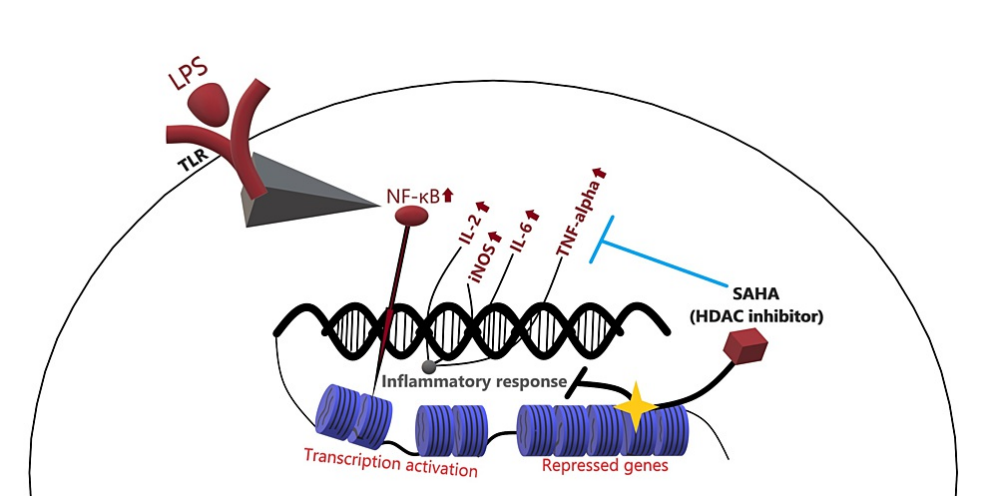
Effects of SAHA and histone acetylation on the LPS-induced inflammatory response in microglial cells SAHA: suberoylanilide hydroxamic acid; LPS: lipopolysaccharide; HDAC: histone deacetylase; TLR: toll-like receptor; NF-κB: nuclear factor kappa-B; TNF-α: tumor necrosis factor alpha; IL-6: interleukin-6; IL-2: interleukin-2; iNOS: inducible nitric oxide synthase

According to the HDAC activity assay results, LPS caused a notable augmentation in the HDAC activity in the N9 microglial cells (P<0.001; Figure 5). Although SAHA at 0.25 and 0.5 μM concentrations failed to decrease the increased LPS-induced total HDAC activity, SAHA inhibited the LPS-induced increase in the total HDAC activity at 1.0 and 1.25 μM concentrations in the N9 microglia (P<0.001; Figures 5, 6).

## Discussion

Our current study revealed that SAHA attenuates the LPS-induced inflammatory response in the microglial cells. SAHA attenuated the increase in TNF-α, IL-6, and IL-1β levels, and improved the decrease in IL-10 levels in the microglial cells. Additionally, SAHA inhibited the increased NF-κB expression against the LPS insult, which was suggested as a response mechanism for TNF-α, IL-6, and IL-1β secretion from microglia.

As the primary innate immune cells of the CNS, microglia are in charge of the continuous surveillance of neuronal homeostasis on external and internal signals [16]. Although the activation of microglia might be a neuroprotective action, the effects of this activation in neurodegenerative disorders are generally related to increased cytokine production [17]. The molecular effects and behavior of microglia are regulated by gene transcription, and therefore, the role of epigenetics in microglia has recently been started to be identified. Contrary to the acute CNS damage, the effects of HDAC inhibition on LPS-treated microglial cells are inconsistent. However, contradictory findings have tried to explain the apoptosis-inducing abilities of HDACs in microglia, which are thought to be in agreement with the results of HDAC inhibition in animal models of acute brain injury [17]. Nevertheless, various histone deacetylase inhibitors have been reported as neuroprotective agents reducing the inflammatory response. Additionally, in vivo and in vitro studies have revealed that HDAC inhibitors modulate the neuroinflammatory response by halting the transcriptional activity of pro-inflammatory genes or proteins [18].

HDAC inhibitors were first tested in the models of neuronal injury and showed potent anti-inflammatory effects and promising results. After understanding these actions on molecular levels, several studies suggested anti-inflammatory mechanisms and pathways that increase neuronal survival. Neuroprotective actions of HDAC inhibitors in Huntington’s disease have already been shown to lead to changes in the microglial gene expression as a response mechanism [19]. Especially evolutionarily conserved mechanisms such as extracellular signal-regulated kinase (ERK), mitogen-activated kinase (MAPK), and c-Jun N-terminal kinase (JNK) and its cellular elements such as NF-κB activation were showed as primary mediators behind these microglial behaviors. The NF-κB promoter activity is a well-known mechanism in LPS-activated microglial cells [20]. This promoter activation gives rise to an increase in several pro-inflammatory cytokines such as TNF-α, IL-6, and IL-1β, and enzymes, also a decrease in IL-10 levels. NF-κB is strongly implicated in the regulation of these enzymes in microglial cells, so neuroprotective actions of several compounds in microglial cells imply this enzymatic action [21].

Jeong et al. demonstrated that the knockdown and selective inhibition of class I HDACs result in the reduced production of cytokines [22]. The HDAC family was first distinguished by their ability to deacetylate histones, but subsequent studies showed that increased gene expression for neuroprotective actions of HDACs is a too simplistic explanation because of the arising repressed genes after SAHA-induced HDAC inhibition. Therefore, more studies on HDAC inhibition on specific proteins and cellular elements are mandatory. If we talk about the anti-inflammatory mechanisms of histone deacetylase inhibitors in microglial cells, NF-κB is the most studied and the first protein that comes to mind. HDAC inhibitors might enhance acetylated NF-κB and cause a decrease in its activity. Furumai et al. showed that the inhibition of HDACs with trichostatin A (TSA), another non-specific inhibitor, results in a decrease in the NF-κB recruitment and RNA polymerase II to the IL-8 promoter, and so a reduction in the NF-κB expression [23]. In line with these studies, SAHA administration decreased NF-κB expression in the N9 microglial cells in our study.

As seen in macrophage cells, microglial cells release harmful cytokines such as TNF-α, IL-6, and IL-1β, and inflammation-resolving cytokines like IL-10, which have attributed beneficial effects on neuron survival [24]. IL-10 has been known to counteract the inflammatory microglial phenotype, which is considered a possible target for anti-inflammatory actions. This phenotype seen in microglial cells was recently shown in the ischemia/reperfusion brain injury model, which increased with histone deacetylase inhibition [25]. Additionally, it was demonstrated that histone deacetylase inhibition suppressed the microglial inflammatory phenotype and cytokines in the LPS-treated microglial cells [26]. In line with these studies, our results demonstrated that there is a decrease in inflammatory cytokine levels, an increase in IL-10 levels, as well as a suppression in NF-κB activation after SAHA administration and HDAC inhibition.

It has already been demonstrated that LPS regulates the expression of pro-inflammatory genes and several members of the histone deacetylase enzyme family [27]. Wu et al. demonstrated histone deacetylase 2 is a crucial element for the LPS-induced inflammatory response in microglia [28]. Additionally, Choi et al. suggested that LPS-induced histone acetylation regulates expression levels of another inflammatory protein, inducible nitric oxide synthase (iNOS) [29]. Our results are coherent with these reports that LPS administration increased the total HDAC activity, and SAHA prevented that increase, which is not surprising.

Our study has some limitations. Firstly, the results of this study are from in vitro experiments. Recent studies have suggested that microglia cell lines differ genetically, transcriptionally, and functionally from primary microglia and ex vivo microglia. Additionally, microglial cell lines obtained from the neonatal or embryonic central nervous system are unlikely to reflect the phenotype of adult or elderly microglia. Despite these limitations, microglia cell lines are suitable for biochemical and molecular approaches. Therefore, primary cultures, in vivo experiments, and clinical studies are necessary to test and verify these results. To our knowledge, this is the first study that shows SAHA-dependent neuroprotection against the commonly used LPS in these cell types, but further studies are needed to understand the effects and detailed mechanism of histone acetylation against oxidative and inflammatory responses.

## Conclusions

With everything taken into account, it can be said that SAHA attenuates the LPS-induced inflammatory response in the N9 microglial cells. The results demonstrated that SAHA treatment decreased harmful cytokine secretion and increased beneficial IL-10 levels. Furthermore, SAHA decreased NF-κB expression in the microglial cells. Additionally, our study suggests that histone acetylation plays a significant role in LPS-induced inflammation; therefore, the regulation of histone acetylation with specific histone deacetylase inhibitors might be a rational perspective for the treatment of neuroinflammation.

## Figures and Tables

**Figure 7:**
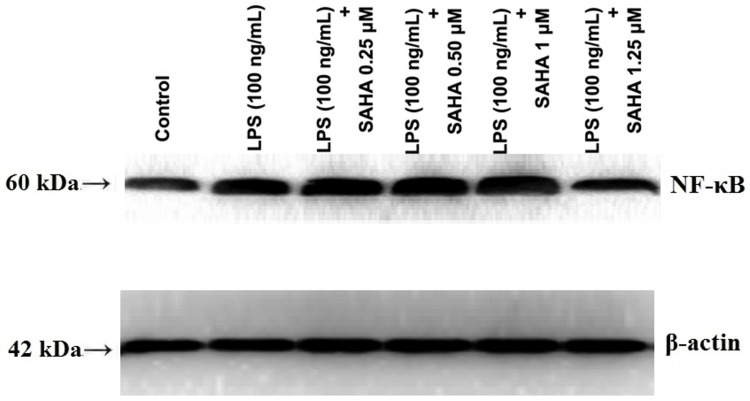
Western blot image and the molecular weight of proteins SAHA: suberoylanilide hydroxamic acid; LPS: lipopolysaccharide; NF-κB: nuclear factor kappa-B
